# Viruliferous rate of small brown planthopper is a good indicator of rice stripe disease epidemics

**DOI:** 10.1038/srep21376

**Published:** 2016-02-22

**Authors:** Dun-Chun He, Jiasui Zhan, Zhao-Bang Cheng, Lian-Hui Xie

**Affiliations:** 1 Fujian Key Lab of Plant Virology, Institute of Plant Virology, Fujian Agriculture and Forestry University, Fuzhou, 350002, China; 2 Institute of Plant Protection, Jiangsu Academy of Agricultural Sciences, Nanjing, 210014, China

## Abstract

*Rice stripe virus* (RSV), its vector insect (small brown planthopper, SBPH) and climatic conditions in Jiangsu, China were monitored between 2002 and 2012 to determine key biotic and abiotic factors driving epidemics of the disease. Average disease severity, disease incidence and viruliferous rate of SBPH peaked in 2004 and then gradually decreased. Disease severity of RSV was positively correlated with viruliferous rate of the vector but not with the population density of the insect, suggesting that the proportion of vectors infected by the virus rather than the absolute number of vectors plays an important role in RSV epidemics and could be used for disease forecasting. The finding of a positive correlation of disease severity and viruliferous rate among years suggests that local infection is likely the main source of primary inoculum of RSV. Of the two main climatic factors, temperature plays a more important role than rainfall in RSV epidemics.

RSV disease is one of the most important viral diseases in East Asia^1^,^2^,^3^, causing up to 50% grain loss^4^,^5^,^6^,^7^ thereby threatening rice production and global food security. The disease was first reported in 1897 in Japan^8^ and has caused several major epidemics in many countries including Japan, China, South Korea, North Korea and Ukraine. For example, in Japan, 13–19% of rice fields were infected by RSV between 1963 and 1967, leading to annual grain losses of ~40,000 metric tons^1^.

In China, RSV was first reported in 1963^9^, spreading rapidly into many rice production areas of the country (>20 provinces). It generally causes 10–20% disease incidence, but more severe epidemics resulting in complete grain loss have been documented in many regions^5^,^10^,^11^,^12^,^13^. Interestingly, the disease was almost absent in Asia in the last decade of the 20^th^ century but re–emerged after 2000 as the most important rice disease in China, particularly in Jiangsu Province, in Japan and South Korea. The resurgence disease induced greater damage compared to its pre–1990 outbreaks, causing 50–100% grain loss in these regions^14^. In 2004 alone, ~1,570,000 hectares of paddy rice in China were infected by the disease, accounting for 79% of total rice production in the country^15^.

Like all other vector–transmitted diseases, the occurrence of RSV and the subsequent development of epidemics result from a complex interaction among five biotic and abiotic factors – the pathogen (RSV), the vector (small brown planthopper, SBPH, *Laodelphax striatellus* Fallén), the host (rice), climate (e.g. temperature, wind, rainfall etc.) and human activity^6^,^16^,^17^,^18^,^19^. Hibino (1996) argued that the main agronomic factors contributing to viral disease epidemics were the widespread use of susceptible varieties, the large–scale cultivation of winter wheat (an alternative host), the sowing and planting time of rice, and the time gap between early and late sowing in rice. Most rice virologists share his view^18^,^20^,^21^,^22^,^23^,^24^. With regard to rice stripe disease, viral source, the density of small brown planthopper (SBPH), rice resistance and temperature were generally considered as the main factors driving the occurrence of the vector and through these viral epidemics^25^.

Kishimoto and Yamada (1986)^26^ proposed the use of SBPH density and viruliferous rate in the overwintering generation to predict RSV epidemics and empirically divided epidemics of RSV into normal–, epidemic– and transitional–level. Viruliferous SBPH density, rice resistance, cultivation time, transplanting time, sowing time, climatic factors^23^,^24^,^27^,^28^,^29^,^30^,^31^,^32^ alone or in combination of 2–3 of these factors have also been proposed as alternative predictors of RSV epidemics by other researchers. However, most previous models were built upon data generated from sprayed fields where disease occurrence and epidemics were under intensive human intervention and hence where some factors, for example, total SBPH density, rice variety, and other cultural practices can be manipulated, but others such as the viruliferous rate of the vector, or temperature cannot. To effectively predict and manage RSV disease, requires knowledge of the biotic and abiotic factors influencing its occurrence and epidemic patterns in both agro–ecosystems experiencing extensive human intervention and semi–natural ecosystems largely lacking direct human input^33^,^34^. In this case, establishing unsprayed fields resembling ecosystems with minimal disease controls as a counterpart to sprayed fields is important to fulfill the ultimate goal of better predicting and managing RSV disease.

Unlike studies of RSV epidemics in the 1960–1970s, in the current experiments co–incident with the current upsurge in RSV, we established a set of sprayed and unsprayed fields in 8 counties in Jiangsu Province where disease and vector were monitored. Disease data gathered from both sprayed and unsprayed fields over a 10–year period of epidemics were evaluated in parallel with vector and climatic data with an objective to develop a lower cost, more effective and ecologically friendly approach for managing RSV disease. The specific goals of the current study were to: 1) understand the temporal dynamics of RSV disease and its vector in one of the largest rice production areas in China; 2) determine the main factors responsible for the epidemics of RSV in the region; and 3) develop a mathematical model to predict the epidemics of RSV based on key factors determining its epidemics.

## Results

### Temporal dynamics in RSV severity, SBPH density and viruliferous rate

**Figure 1:**
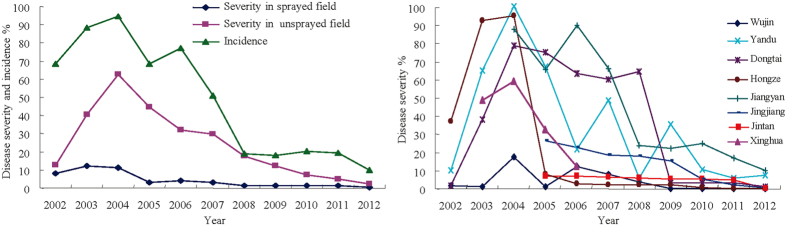
Temporal dynamics of RSV epidemics between 2002 and 2012 in Jiangsu Province: 1) Disease severity and incidence (% of acreage infected) across the eight counties; 2) Disease severity in unsprayed fields by county.

**Figure 2:**
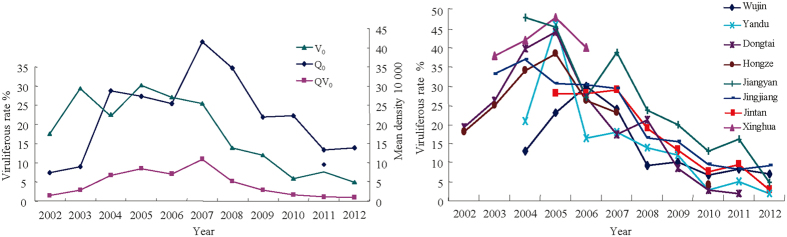
Temporal dynamics of density and viruliferous characteristics of SBPH between 2002 and 2012 in Jiangsu Province: 1) total SBPH density, viruliferous rate and viruliferous SBPH density at overwintering time across the eight counties; 2) viruliferous rate at overwintering time by county. Note: Q_0_, V_0_ and QV_0_ are total SBPH density, viruliferous rate and viruliferous SBPH density at overwintering stage, respectively.

In unsprayed fields, rice stripe disease was observed only sporadically before 2001. The average disease severity rose quickly from ~12% in 2002 to peak at ~63% in 2004 before gradually decreased to ~2% in 2012 (Fig. 1, Table S1). Though varying in scale, similar patterns of temporal dynamics in disease severity were observed among the regions (8 counties, Fig. 1) and between sprayed and unsprayed fields. The percentage of rice acreage infected by RSV (disease incidence) showed a similar temporal trend as disease severity (Fig. 1). Total SBPH density and viruliferous rate varied greatly among years and regions (Fig. 2, Table S2). On the other hand, there was less spatiotemporal variation in viruliferous SBPH density (Fig. 2). Over the period of the study, viruliferous rate peaked at approximately the same time as disease severity but viruliferous and total SBPH density peaked in 2007, lagging three years behind disease severity.

### Associations of rice stripe disease with SBPH traits

Rice stripe disease severity in both sprayed and unsprayed fields in any particular year was positively correlated with viruliferous rate in the preceding, current and following years at all epidemic stages but not with viruliferous or total SBPH density (Table 1). Though the overall patterns were similar across epidemic stages and field treatments, the association between disease severity and viruliferous rate in unsprayed fields was stronger than that in sprayed fields and that in the first generation was higher than that in overwintering vectors. The correlations between disease severity of the three years (preceding, current and following) and total SBPH density at the peak stage in those years were significantly negative but no associations were found between the two parameters at other epidemic stages. The relationship between disease severity and viruliferous rate of overwintering SBPH fitted a logistic model (Table 2) as expressed by ln (y∙100) = 4.2–0.1861/x, where y is the expected disease severity and x is the viruliferous rate of overwintering SBPH.

### Associations of rice stripe disease with climatic factors

Among relevant climate factors, mean temperature in May alone was significantly and negatively correlated with rice stripe disease severity in unsprayed fields (Table 3). In sprayed fields, disease severity was also significantly negatively correlated with mean temperatures in May and weakly so with May minimum temperatures. Temperatures in other months and rainfall showed no correlation with disease severity.

### Associations of disease severity among epidemic years

In unsprayed fields, rice stripe disease severity in any given year was positively and significantly correlated with that of preceding and current year levels in sprayed fields and the percentage of diseased fields in that year (Table 4). Furthermore, the mean disease severity in unsprayed fields in the eight counties was also positively correlated with that in sprayed fields (r_10_ = 0.760, p = 0.007) and the percentage of diseased fields (r_10_ = 0.850, p = 0.001).

### Associations among SBPH traits

Total SBPH density, viruliferous rate and viruliferous SBPH density in the first generation were positively correlated with those in the overwintering generation (Table 5). Though negative, the correlation between total SBPH density and viruliferous rate was not significant.

### Associations between SBPH and climatic conditions

Most correlations between viruliferous rate and temperature were not significant both in the overwintering and first generation of SBPH. With a few exceptions, total SBPH density in the overwintering and first generation was positively correlated with maximum temperature and negatively correlated with minimum temperature in each month (Table 6).

## Discussion

Viruliferous population density has been widely used as a management indicator for insect–transmitted viral diseases^32^,^35^. However, our analysis shows that the RSV disease severity does not correlate with viruliferous SBPH density in either sprayed or unsprayed fields. Rather, it is positively correlated with viruliferous rate, consistent with previous studies in other rice viral diseases^23^,^36^. This result suggests that, the proportion, rather than the absolute size of the SBPH population infected by the virus, plays the decisive role in RSV epidemics. This counter–intuitive result is likely to result from a trade–off between the role of infected SBPH insects in disease epidemics and their own reproduction. While infection of insects by the virus is a pre–requirement for its transmission, and therefore disease epidemics, it also reduces the survival and reproductive ability of the insects themselves^37^,^38^. This result also suggests that viruliferous rate could be a good indicator of RSV disease epidemics and management such that the equation ln (y∙100) = 4.2–0.1861/x [where y is the expected disease severity and x is the viruliferous rate of overwintering SBPH] could be used for disease forecasting. Whether such a relationship is only relevant to plant viruses that propagate vertically within insect vectors and transmitted to their progenies in a persistent manner such as RSV^39^,^40^, or is ubiquitous in all insect–transmitted plant viruses needs further study.

The observation that the proportion, rather than the absolute size of the SBPH population infected by the virus plays the decisive role in RSV disease epidemics is also supported by negative associations of total SBPH density with RSV severity and viruliferous rate. Viruliferous rates of SBPH in all epidemic stages were significantly and positively correlated with rice stripe disease severity in the preceding, current and following year in sprayed and unsprayed fields, which suggests that the viruliferous rate in a given year likely results from disease epidemics in the preceding year. The preceding epidemic is the primary viral source for new infection, as indicated by a positive correlation between disease severities among years. Under a constant viral source, increasing insect density reduces the chance of any individual insect becoming infected, leading to a negative correlation between total SBPH density and viruliferous rate. Furthermore, large numbers of uninfected insects competing with infected ones to feed on a newly established crop, reduce the chance of viruliferous insects transmitting the virus and hence causing disease epidemics.

The positive correlation between disease severities among years indicates that local infection is the main source of primary inoculum, as reported earlier^41^,^42^,^43^,^44^. In this scenario, it is expected that disease severity in the fields would change in a consistent direction. Unexpectedly, RSV severity in the areas studied showed a bell distribution, increasing from 2002 to 2004 and then declining. RNA viruses such as RSV are characterized by high mutation rates attributing to their lack of proofing mechanisms in genome duplication^45^,^46^,^47^,^48^,^49^,^50^. Genetic meltdown leading to changes in infectivity (of either insect or host plant) due to rapid accumulation of deleterious mutations^45^,^46^,^47^,^48^,^49^,^50^,^51^ could partially contribute to the switch of increasing epidemics to decreasing epidemics over the observed time scale. However, the observed changing epidemic pattern in the current study is most likely due to some form of human intervention. In this part of China SBPH overwinters mainly on wheat. Since the onset of the current epidemic cycle, local governments have launched an initiative to eradicate SBPH overwintering sites by reducing or stopping wheat cultivation in the region. Virus acquisition of SBPH depends on the probability of transovarial transmission or acquisition from diseased host plants. Viruliferous rates of SBPH will decrease gradually without any viral source supplement^52^,^53^.

With the exception of the first generation of SBPH density (Q_1_), no associations were detected between rainfall and insect parameters. On the other hand, many correlations between temperature and insect parameters were significant. These results indicate that temperature plays a more important role than rainfall in the survival and reproduction of SPBH. The optimum temperature for survival and reproduction of SBPH is about 25 °C and higher winter temperatures are apparently favorable for the overwintering of SBPH^54^, as supported by the positive correlation between mean temperature and overwintering SBPH density (Q_0_) (Table 6). In addition, winter temperature also alters SBPH feeding behavior with warmer winters enhancing feeding activity as indicated by the positive correlation between temperature and viruliferous population density of overwintering SBPH (QV_0_). However, the impact of temperature on SBPH survival, reproduction and feeding behavior is mainly restricted to the overwintering stage. In other phases of life cycle, the contribution of thermal fluctuation to the survival, reproduction and feeding behavior is negligible as indicated by a lack of correlation involving local temperature with SBPH density of peak stage (Q_max_), Q_1_, viruliferous rate (V_1_) or viruliferous population density (QV_1_) of first generation SBPH.

In this study, we collected data from both sprayed and unsprayed rice fields. Disease in unsprayed fields is less influenced by human activities, and therefore more closely resembles disease and epidemic occurrence in semi–natural ecosystems. Such areas are a potential source of primary inoculum for a new epidemic in the coming season. Comparing the temporal dynamics of RSV in sprayed and unsprayed fields is important as a means of untangling some of the differences between agricultural and more natural systems that drive the epidemiology of this disease. Such an approach could be of value if applied to studies of other plant viral diseases.

## Materials and Methods

**Figure 3:**
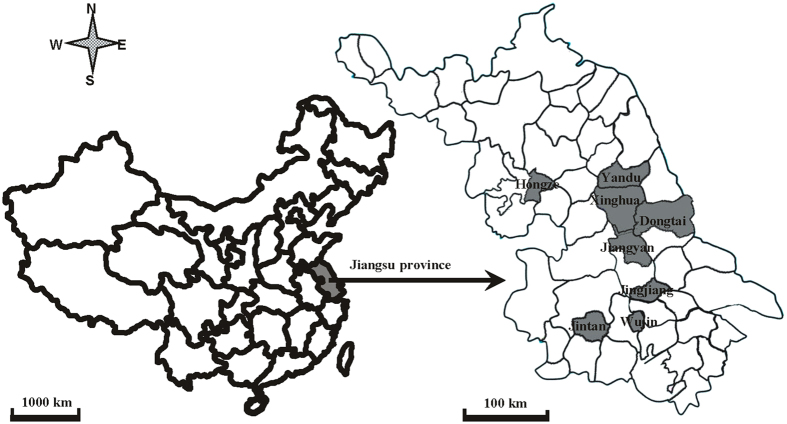
Map showing the geographical distribution of the eight main rice production counties in Jiangshu Province and the sites of the sprayed and unsprayed fields monitored in the survey. Adobe Illustrator Artwork 17.0 software was used to create the map.

Rice stripe disease severity was monitored annually over the period 2002–2012 inclusively in a total of 4000 insecticide–sprayed (chemical used) paddy fields distributed across the eight major rice–growing counties of Jiangsu Province, China (Fig. 3). Each county was represented by five towns and each town was represented by five villages. In each village, 20 paddy fields each at least 667 m^2^ in size were randomly selected for the experiment. An identical sampling protocol was used over the entire 10 year period with disease severity in each field being determined from 200 plants distributed at five points determined using a random spatial sampling approach^55^. RSV severity at each sampling point was recorded using a five–grade system (<5%, 5%–10%, 10%–20%, 20%–50% and >50%) as described previously^55^. The disease assessment took place at the same phenological stage (late tillering state) every year – a stage that experience had shown to provide a reliable estimate of epidemic development. An unsprayed (no insecticide application) field with a size of ~667 m^2^ was used as a control in each village with disease severity being assessed in the same way as for the sprayed fields. The mean disease severity (MDSev) in each county was estimated as:


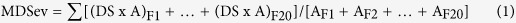


where DS is the mid–range figure of each disease severity grade (i.e. 2.5, 7.5, 15, 35, 75%), A is the area of the field, and Fn is the field number. The percentage of infected fields was determined by dividing the number of fields in each county infected by virus by the total number of fields.

Total SBPH density and viruliferous rate of SBPH were recorded during the overwintering (December to March) and first generation phases (March to May) in wheat fields and at the peak phase (June) in the rice paddy fields. Total SBPH density was determined by the plant–flapping (on the roots in seedling stage or flag leaves in earing stage) approach using a porcelain plate (40 cm × 30 cm) as described previously^56^. The number of insects in each sampling point was counted from 20 porcelain plates and five sampling points each with 300 plants in a field were included in density analysis. SBPH viruliferous rate of each county in each year was determined by Dot–ELISA^57^ using 98 insects randomly selected from the total collection. RSV monoclonal antibody was prepared by our laboratory using Goat–anti–Mouse IgG, chromomeric substrate and other reagents were purchased from the Sigma Company (No.398, Huaihai Road, Shanghai). The viruliferous rate across eight counties in each year was calculated as:


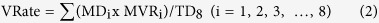


where MD is the total SBPH density, MVR is the SBPH viruliferous rate of each county, TD is the total SBPH density across eight counties and i is the random order of these eight counties.

Climate data including monthly mean, minimum and maximum temperature and daily mean rainfall from December to May for each year were obtained from local weather stations in the eight counties (Table S3).

Disease severity, SBPH density and viruliferous rate in sprayed and unsprayed fields were tabulated over the epidemic years for each individual county as well as for the combined data from different counties. The association between and among disease severity, total SBPH density, viruliferous rate, viruliferous SBPH density and climatic parameters including strength, direction and quantity of the associations were analyzed using Spearman’s correlation^58^ taking the variables in each year and county as random parameters. In these analyses, all variables were ranked and correlation coefficients were calculated according to the order of the variable. Spearman correlation coefficient was calculated as:


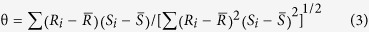


where *R*_*i*_ and *S*_*i*_ are the rank of element *i* in the independent and dependent variables and 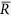 and 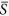 are the mean of independent and dependent variables, respectively.

In association analyses, it is necessary to guard against non–independent elements among the variables as they have the potential to affect the reliability of the extent and direction of estimates. However, by converting data to ranks (1,2,3,4, … etc.) and estimating correlation coefficients on these ranked values the Spearman correlation is less dependent on the randomness of variable elements^59^. Therefore, because severity, and possible driving forces such as SBPH density of RSV epidemics, may not be independent from year to year, we used this statistic to evaluate the associations between disease, vector and climatic data.

## Additional Information

**How to cite this article**: He, D.-C. *et al.* Viruliferous rate of small brown planthopper is a good indicator of rice stripe disease epidemics. *Sci. Rep.* **6**, 21376; doi: 10.1038/srep21376 (2016).

## Supplementary Material

Supplementary Information

## Figures and Tables

**Table 1:** Associations of RSV severity with density and viruliferous characters of SBPH in Jiangsu Province (Data summed across all sites).

		Severity in unsprayed fields			Severity in sprayed fields		
		Preceding year	Current year	Following year	Preceding year	Current year	Following year
Preceding year	Q_0_	−0.076	−0.147	−0.117	0.098	0.067	0.133
	V_0_	0.652**	0.528**	0.426**	0.474**	0.346*	−0.008
	Q_1_	0.014	−0.107	−0.118	−0.012	−0.056	0.012
	V_1_	0.814**	0.756**	0.684**	0.646**	0.510**	0.195
	Q_max_	−0.447**	−0.622**	−0.723**	−0.465**	−0.568**	−0.556**
	QV_0_	0.139	0.063	0.057	0.267	0.238	0.237
	QV_1_	0.190	0.254	0.458**	0.151	0.197	0.196
Current year	Q_0_	−0.015	−0.076	−0.147	0.164	0.098	0.067
	V_0_	0.601**	0.652**	0.528**	0.645**	0.474**	0.346*
	Q_1_	0.187	0.014	−0.107	0.180	−0.012	−0.056
	V_1_	0.758**	0.814**	0.756**	0.775**	0.646**	0.510**
	Q_max_	−0.356*	−0.447**	−0.622**	−0.332*	−0.465**	−0.568**
	QV_0_	0.099	0.139	0.063	0.355*	0.267	0.238
	QV_1_	0.246	0.190	0.254	0.278	0.151	0.197
Following year	Q_0_	−0.043	−0.015	−0.076	0.169	0.164	0.098
	V_0_	0.456**	0.601**	0.652**	0.640**	0.645**	0.474**
	Q_1_	0.216	0.187	0.014	0.259	0.180	−0.012
	V_1_	0.401*	0.758**	0.814**	0.824**	0.775**	0.646**
	Q_max_	−0.331*	−0.356*	−0.447**	−0.239	−0.332*	−0.465**
	QV_0_	0.012	0.099	0.139	0.239	0.355*	0.267
	QV_1_	0.291	0.246	0.190	0.479**	0.278	0.151

Q_0_: overwintering SBPH density, V_0_: viruliferous rate of overwintering SBPH, Q_1_: first generation SBPH density, V_1_: viruliferous rate of first generation SBPH, Q_max_: SBPH density of peak stage, QV_0_: viruliferous population density of overwintering SBPH (Q_0_ × V_0_), QV_1_: viruliferous population density of first generation SBPH (Q_1_ × V_1_).

**significant at p = 0.01; *significant at p = 0.05.

**Table 2:** The test statistic of S−curve estimation.

Adjusted R^2^	F	Sig.	T		Sig.	
			1/x	Constant	1/x	Constant
0.829	49.410	0.000	−7.029	17.807	0.000	0.000

**Table 3:** Associations of rice stripe disease severity with monthly temperature and rainfall in SBPH overwintering and rice growing seasons (December to May).

Severity	Month	Mean T (C)	Min T (C)	Max temperature	Mean rainfall daily
Unsprayed field	Dec	0.026	0.066	0.044	−0.159
	Jan	−0.012	0.045	0.022	0.161
	Feb	0.084	0.017	0.020	−0.082
	Mar	−0.066	−0.045	0.015	−0.084
	Apr	−0.238	−0.030	0.007	0.051
	May	−0.389*	−0.228	−0.119	0.033
Sprayed field	Dec	0.048	−0.078	0.137	−0.065
	Jan	0.147	−0.046	0.180	0.181
	Feb	0.107	−0.121	0.069	0.01
	Mar	0.034	−0.138	0.168	0.030
	Apr	−0.122	−0.049	0.133	0.080
	May	−0.431**	−0.274	−0.035	0.077

**significant at p = 0.01; *significant at p = 0.05.

**Table 4:** Associations of disease severity among epidemic years.

	Severity in unsprayed field in preceding year	Severity in sprayed field in current year	Severity in unsprayed field in current year	Disease incidence in current year
Severity in sprayed field in preceding year	0.722**	0.844**	0.691**	0.618**
Severity in unsprayed field in preceding year		0.538**	0.735**	0.417**
Severity in sprayed field in current year			0.722**	0.778**
Severity in unsprayed field in current year				0.583**

**significant at p = 0.01.

**Table 5:** Associations between density and viruliferious characteristics of SBPH populations.

	V_0_	Q_1_	V_1_	Q_max_	QV_0_	QV_1_
Q_0_	−0.186	0.641**	−0.419*	0.113	0.871**	0.386*
V_0_		−0.119	0.859**	−0.400*	0.283	0.272
Q_1_			−0.108	0.235	0.555**	0.544**
V_1_				−0.222	−0.013	0.423*
Q_max_					−0.025	−0.127
QV_0_						0.458**

Q_0_: overwintering SBPH density, V_0_: viruliferous rate of overwintering SBPH, Q_1_: first generation SBPH density, V_1_: viruliferous rate of first generation SBPH, Q_max_: SBPH density of peak stage, QV_0_: viruliferous population density of overwintering SBPH (Q_0_ × V_0_), QV_1_: viruliferous population density of first generation SBPH (Q_1_ × V_1_).

**significant at p = 0.01; *significant at p = 0.05.

**Table 6:** Associations of local temperature and rainfall with density and viruliferous characteristics of the SBPH population.

	Month	Mean T (C)	Min T (C)	Max temperature	Mean rainfall daily
V_0_	Dec	0.048	0.235	−0.128	−0.097
	Jan	0.057	0.293	−0.168	0.375*
	Feb	0.032	0.213	−0.160	0.020
	Mar	0.163	0.164	−0.110	−0.042
Q_0_	Dec	0.445**	−0.453**	0.537**	−0.184
	Jan	0.320*	−0.369*	0.464**	0.007
	Feb	0.226	−0.405**	0.575**	−0.045
	Mar	0.307*	−0.495**	0.563**	−0.131
QV_0_	Dec	0.338*	−0.014	0.125	0.106
	Jan	0.423**	0.019	0.157	0.221
	Feb	0.313*	0.112	0.133	0.209
	Mar	0.320*	−0.172	0.316	0.078
V_1_	Dec	−0.108	0.062	−0.006	0.024
	Jan	−0.147	0.054	−0.073	0.375*
	Feb	−0.021	0.062	−0.326	0.117
	Mar	−0.114	−0.074	−0.163	0.099
	Apr	−0.198	0.240	−0.036	0.218
	May	−0.441**	−0.200	−0.404*	0.291
Q_1_	Dec	0.367*	−0.326*	0.426**	−0.302*
	Jan	0.147	−0.258	0.332*	−0.234
	Feb	0.1396	−0.371*	0.435**	−0.347*
	Mar	0.163	−0.355*	0.334*	−0.434**
	Apr	−0.089	−0.482**	0.353*	−0.317*
	May	0.207	−0.428**	0.385*	−0.212
QV_1_	Dec	0.303	−0.337	0.394*	−0.128
	Jan	0.247	−0.135	0.228	0.126
	Feb	0.176	−0.137	0.157	−0.090
	Mar	0.225	−0.181	0.206	−0.219
	Apr	0.112	−0.053	0.336	−0.046
	May	0.065	−0.455**	0.121	0.025
Q_max_	Dec	−0.064	0.009	−0.035	0.081
	Jan	−0.285	−0.059	−0.031	−0.089
	Feb	−0.086	−0.073	0.079	0.032
	Mar	0.016	−0.024	−0.085	0.041
	Apr	−0.187	−0.147	−0.109	−0.056
	May	0.222	0.033	0.119	0.198

Q_0_: overwintering SBPH density, V_0_: viruliferous rate of overwintering SBPH, Q_1_: first generation SBPH density, V_1_: viruliferous rate of first generation SBPH, Q_max_: SBPH density of peak stage, QV_0_: viruliferous population density of overwintering SBPH (Q_0_ × V_0_), QV_1_: viruliferous population density of first generation SBPH (Q_1_ × V_1_).

**significant at p = 0.01; *significant at p = 0.05.
