# Supplementary material for: Viruliferous rate of small brown planthopper is a good indicator of rice stripe disease epidemics
Source: Sci Rep. 2016 Feb 22;6:21376. doi: 10.1038/srep21376 (PMC4761966; doi:10.1038/srep21376)
Supplement: Supplementary Information [file srep21376-s1.pdf]

Viruliferous rate of small brown planthopper is a good indicator of rice stripe disease epidemics

Dun-Chun He[1], Jiasui Zhan[1], Zhao-Bang Cheng[2*], Lian-Hui Xie[1*]

[1]Fujian Key Lab of Plant Virology, Institute of Plant Virology, Fujian Agriculture and Forestry University, Fuzhou, 350002, China; [2]Institute of Plant Protection, Jiangsu Academy of Agricultural Sciences, Nanjing, 210014, China

[*]Corresponding authors: Cheng Z B (E-mail: onlyone8501@126.com) and Xie L H (E-mail: xielh@fafu.edu.cn)

Table S1 RSV disease severity and incidence in sprayed and unsprayed fields between 2002-2012

County	year	Rice area (667 ha)	Sprayed fields area (667 ha)					RSV severity in unsprayed fields
			<5%	5%-10%	10%-20%	20%-50%	>50%	
Wujin	2002	46.930	19.560	9.150	3.290	0.370	0.000	1.710
Wujin	2003	38.450	18.200	6.100	5.500	0.600	0.000	1.180
Wujin	2004	35.885	32.060	5.710	3.490	0.270	0.000	17.430
Wujin	2005	34.720	13.430	2.400	0.600	0.300	0.000	1.120
Wujin	2006	31.800	20.640	5.300	1.060	0.280	0.000	12.340
Wujin	2007	28.200	23.560	2.590	0.370	0.370	0.000	7.860
Wujin	2008	26.100	9.850	1.400	0.000	0.000	0.000	3.590
Wujin	2009	25.500	8.520	0.300	0.000	0.000	0.000	0.000
Wujin	2010	23.500	4.016	0.000	0.000	0.000	0.000	0.000
Wujin	2011	20.120	4.014	0.000	0.000	0.000	0.000	0.000
Wujin	2012	19.020	3.630	0.000	0.000	0.000	0.000	0.000
Jintan	2005	32.000	8.010	0.000	0.000	0.000	0.000	6.730
Jintan	2006	31.900	7.690	0.000	0.000	0.000	0.000	-
Jintan	2007	31.700	7.680	0.000	0.000	0.000	0.000	6.350
Jintan	2008	32.000	6.540	0.160	0.000	0.000	0.000	5.810
Jintan	2009	31.200	4.320	0.000	0.000	0.000	0.000	5.460
Jintan	2010	32.000	1.870	0.000	0.000	0.000	0.000	5.100
Jintan	2011	30.720	0.060	0.000	0.000	0.000	0.000	4.900
Jintan	2012	30.510	0.000	0.000	0.000	0.000	0.000	0.000
Xinghua	2003	130.900	52.600	20.000	37.000	18.000	3.300	48.290
Xinghua	2004	131.900	27.870	45.630	41.670	13.770	2.960	58.620
Xinghua	2005	134.800	121.610	9.300	3.190	0.570	0.130	32.630
Xinghua	2006	134.500	132.900	1.520	0.070	0.000	0.000	12.310
Yandu	2002	56.000	13.700	18.000	3.000	1.000	0.000	9.900
Yandu	2003	53.000	6.800	18.200	10.000	3.400	1.600	64.600
Yandu	2004	60.000	13.000	15.500	9.500	6.500	4.500	100.000
Yandu	2005	60.380	2.300	1.800	0.500	0.000	0.000	66.700
Yandu	2006	61.000	35.000	15.000	2.600	0.000	0.000	21.400
Yandu	2007	60.360	39.200	5.000	2.400	0.300	0.200	48.300
Yandu	2008	63.000	0.340	0.050	0.025	0.010	0.000	5.800
Yandu	2009	62.000	11.970	0.270	0.085	0.005	0.000	35.400
Yandu	2010	62.000	0.700	0.000	0.000	0.000	0.000	10.400
Yandu	2011	62.000	6.520	0.300	0.050	0.000	0.000	5.600
Yandu	2012	62.000	0.390	0.000	0.000	0.000	0.000	7.500
Dongtai	2002	34.190	14.670	1.760	0.650	0.040	0.000	1.330
Dongtai	2003	33.590	18.670	3.880	1.240	0.250	0.000	37.800
Dongtai	2004	38.960	12.470	16.780	3.480	0.520	0.250	78.540

Dongtai	2005	38.600	27.430	0.490	0.000	0.000	0.000	74.490
Dongtai	2006	40.300	21.330	0.270	0.000	0.000	0.000	63.190
Dongtai	2007	38.890	26.800	0.100	0.000	0.000	0.000	60.050
Dongtai	2008	40.230	12.170	0.000	0.000	0.000	0.000	64.280
Dongtai	2009	40.000	10.980	0.000	0.000	0.000	0.000	3.300
Dongtai	2010	40.000	8.320	0.000	0.000	0.000	0.000	3.000
Dongtai	2011	40.000	8.880	0.000	0.000	0.000	0.000	3.000
Dongtai	2012	48.000	4.010	0.000	0.000	0.000	0.000	1.000
Hongze	2000	35.000	21.500	2.800	3.200	1.600	0.270	19.000
Hongze	2001	35.000	18.000	3.600	3.500	1.700	0.400	23.000
Hongze	2002	35.000	6.000	10.500	10.000	6.000	0.500	37.000
Hongze	2003	35.000	10.000	8.000	6.000	8.000	1.200	92.000
Hongze	2004	35.000	13.000	9.300	4.800	6.500	0.900	95.000
Hongze	2005	40.500	16.700	0.340	0.000	0.000	0.000	7.800
Hongze	2006	41.500	2.200	0.000	0.000	0.000	0.000	2.800
Hongze	2007	42.500	2.000	0.000	0.000	0.000	0.000	2.200
Hongze	2008	42.500	1.900	0.000	0.000	0.000	0.000	2.000
Hongze	2009	42.500	1.300	0.000	0.000	0.000	0.000	1.900
Hongze	2010	42.500	1.000	0.000	0.000	0.000	0.000	0.500
Hongze	2011	42.500	0.800	0.000	0.000	0.000	0.000	0.200
Hongze	2012	42.500	0.100	0.000	0.000	0.000	0.000	0.100
Hongze	2013	42.500	0.000	0.000	0.000	0.000	0.000	0.000
Jiangyan	2004	55.000	24.390	25.460	0.600	1.200	0.350	87.500
Jiangyan	2005	58.000	23.430	22.700	0.455	0.224	0.058	65.500
Jiangyan	2006	58.000	19.880	23.360	0.105	0.127	0.046	89.670
Jiangyan	2007	58.000	19.130	18.110	0.194	0.019	0.097	66.050
Jiangyan	2008	57.000	14.210	5.360	0.111	0.049	0.011	23.750
Jiangyan	2009	49.500	6.140	2.890	0.004	0.007	0.003	22.250
Jiangyan	2010	49.000	4.510	6.700	0.011	0.026	0.025	24.550
Jiangyan	2011	49.500	4.700	4.930	0.025	0.010	0.000	17.000
Jiangyan	2012	50.160	0.003	0.081	0.000	0.000	0.000	10.250
Jingjiang	2003	32.340	15.680	9.960	7.010	1.170	0.380	-
Jingjiang	2004	34.470	35.590	0.610	0.000	0.000	0.000	-
Jingjiang	2005	34.930	25.866	6.820	0.530	0.004	0.000	26.500
Jingjiang	2006	35.230	20.490	10.150	0.580	0.019	0.008	22.400
Jingjiang	2007	35.150	16.938	0.160	0.110	0.002	0.000	18.600
Jingjiang	2008	34.560	10.770	0.810	0.390	0.030	0.000	18.000
Jingjiang	2009	33.780	9.030	0.630	0.230	0.000	0.000	15.400
Jingjiang	2010	33.290	30.680	0.860	0.410	0.000	0.000	5.200
Jingjiang	2011	32.860	26.230	0.410	0.050	0.000	0.000	2.090
Jingjiang	2012	32.510	17.510	0.000	0.000	0.000	0.000	0.630

Table S2 Population density and viruliferous rate of small brown planthopper in each of eight counties

County	Year	Population density of overwintering SBPH (10000)	Viruliferous rate of overwintering SBPH %	Population density of First generation SBPH (10000)	Population density of peak stage (10000)	Peak stage (dates)
Dongtai	2002	0.61	19.20	15.23	7.39	31/5-9/6
Dongtai	2003	0.35	26.25	24.22	6.12	4/6-12/6
Dongtai	2004	3.12	39.65	62.26	9.69	4/6-10/6
Dongtai	2005	1.56	44.25	27.75	18.86	2/6-8/6
Dongtai	2006	0.95	27.27	68.81	22.21	3/6-11/6
Dongtai	2007	1.58	17.23	84.19	88.30	29/5-5/6
Dongtai	2008	0.89	21.30	29.95	71.55	3/6-7/6
Dongtai	2009	1.28	8.50	62.02	92.28	4/6-12/6
Dongtai	2010	0.80	3.00	79.72	127.67	8/6-18/6
Dongtai	2011	0.39	2.00	53.73	143.85	1/6-9/6
Dongtai	2012	0.82	-	-	-	-
Wujin	2004	14.70	13.10	162.30	3.34	29/5-24/6
Wujin	2005	9.12	23.00	108.20	5.44	29/5-5/6
Wujin	2006	5.46	29.90	95.60	90.24	29/5-3/6
Wujin	2007	3.00	24.00	77.60	135.68	28/5-3/6
Wujin	2008	2.01	9.30	72.80	178.80	1/6-5/6
Wujin	2009	4.98	10.20	58.30	70.40	1/6-5/6
Wujin	2010	12.02	6.54	66.20	-	8/6-15/6
Wujin	2011	2.80	8.10	50.60	-	-
Wujin	2012	4.29	6.92	-	-	-
Jintan	2005	7.00	-	6.24	28.74	25/5-1/6
Jintan	2006	-	28.00	10.66	35.77	22/5-30/5
Jintan	2007	9.25	29.00	59.69	95.20	21/5-27/5
Jintan	2008	1.98	18.80	42.43	46.83	15/5-21/5
Jintan	2009	4.80	13.40	5.47	15.87	22/5-28/5
Jintan	2010	1.09	7.69	8.49	7.35	1/6-9/6
Jintan	2011	0.80	9.62	-	-	-
Jintan	2012	0.21	2.78	-	-	-
Xinghua	2003	0.32	38.00	3.85	7.23	21/5-29/5
Xinghua	2004	0.70	42.00	12.10	19.20	25/5-2/6
Xinghua	2005	1.10	48.00	22.60	21.50	20/5-26/5
Yandu	2002	0.28	-	2.13	2.19	1/6-10/6
Yandu	2003	0.08	-	0.50	0.58	10/6-17/6
Yandu	2004	0.86	20.80	32.40	41.50	2/6-10/6

Yandu	2005	1.28	46.20	37.30	47.50	2/6-12/6
Yandu	2006	3.30	16.50	50.10	48.50	5/6-10/6
Yandu	2007	8.89	18.00	107.46	110.40	27/5-13/6
Yandu	2008	22.80	14.00	300.69	214.30	27/5-14/6
Yandu	2009	2.83	12.00	62.70	123.30	3/6-13/6
Yandu	2010	2.32	3.00	93.00	228.40	10/6-18/6
Yandu	2011	2.90	-	-	-	-
Yandu	2012	2.30	2.00	-	-	-
Hongze	2002	1.20	18.00	2.76	3.34	2/6-5/6
Hongze	2003	1.40	25.00	3.68	33.88	3/6-7/6
Hongze	2004	0.18	34.00	6.32	33.90	3/6-7/6
Hongze	2005	0.18	38.50	29.40	47.49	3/6-7/6
Hongze	2006	0.23	26.30	7.50	90.37	6/6-12/6
Hongze	2007	0.41	23.00	14.50	124.00	1/6-5/6
Hongze	2008	0.22	-	20.70	400.00	4/6-6/6
Hongze	2009	0.21	-	19.60	412.20	3/6-7/6
Hongze	2010	0.05	4.00	14.10	359.80	15/6-19/6
Hongze	2011	0.05	-	14.70	101.50	10/6-14/6
Hongze	2012	0.08	-	-	-	-
Jiangyan	2004	0.56	47.90	22.50	15.60	25/5-11/6
Jiangyan	2005	0.25	45.50	78.50	13.04	23/5-15/6
Jiangyan	2006	1.28	27.20	125.00	5.74	25/5-15/6
Jiangyan	2007	3.36	38.97	217.68	78.52	23/5-11/6
Jiangyan	2008	0.97	23.65	33.08	75.29	22/5-13/6
Jiangyan	2009	1.06	20.00	84.28	18.78	23/5-14/6
Jiangyan	2010	1.00	13.00	23.52	5.76	24/5-18/6
Jiangyan	2011	1.60	16.25	178.02	17.23	24/5-14/6
Jiangyan	2012	1.47	4.67	-	-	-
Jingjiang	2003	2.38	-	51.73	-	-
Jingjiang	2004	-	36.80	107.64	45.60	29/5-30/5
Jingjiang	2005	4.56	30.77	39.27	33.78	31/5-1/6
Jingjiang	2006	5.58	30.20	109.74	58.30	1/6-2/6
Jingjiang	2007	9.56	29.50	188.55	19.24	24/5-25/5
Jingjiang	2008	1.73	16.50	104.42	142.28	24/5-25/5
Jingjiang	2009	4.24	15.40	60.07	87.60	28/5-29/5
Jingjiang	2010	2.26	9.50	30.13	59.80	4/6-5/6
Jingjiang	2011	3.22	8.30	30.30	198.23	8/6-10/6
Jingjiang	2012	2.94	9.26	-	-	-

Table S3 Temperature and rainfall data in the eight counties from 2002 to2012

County	Year	Dec				Jan				Feb				Mar				Apr				May			
		Mean T (C)	Min T (C)	Max T (C)	Mean rainfall daily	Mean T (C)	Min T (C)	Max T (C)	Mean rainfall daily	Mean T (C)	Min T (C)	Max T (C)	Mean rainfall daily	Mean T (C)	Min T (C)	Max T (C)	Mean rainfall daily	Mean T (C)	Min T (C)	Max T (C)	Mean rainfall daily	Mean T (C)	Min T (C)	Max T (C)	Mean rainfall daily
Wujin	2002	5.6	-4.2	16.3	2.67	5.4	19.9	-2.8	1.07	7.6	-1	14.8	1.28	12.26	2.2	17.2	2.38	16.4	6.3	29.3	2.22	19.2	12.7	30.1	7.89
Wujin	2003	5	-3.5	17.8	0.8	2.7	-6.8	16	1.35	5.9	-3.4	18.6	2.12	9.5	0.9	28.2	3.65	15.1			3.33	20	10.2	29.8	1.04
Wujin	2004	7.2	-5.9	16.8	1.33	3.4	-6.5	8.8	2.18	8.1	-3.1	19.1	1.55	9.4	0.7	26.3	1.24	16.3	2.1	33.4	3.64	20.8	9.7	34.4	3.42
Wujin	2005	7.2	-7.1	14.3	1.33	1.9	-7.2	8.9	1.23	3.2	-4.6	14.1	2.94	8.9	-2.7	24.1	1.33	18	7.6	33.6	4.3	20.7	12.8	29.7	3.07
Wujin	2006	3.9	-3.8	16	0.67	4.4	-5.1	18.2	4.07	4.5	-4.6	19	2.55	11.1	-0.3	22.8	0.72	16.6	4.7	28.4	4.3	20.9	12.6	31.5	2.59
Wujin	2007	7.1	-2.8	16.1	1.36	4	-3.4	13.1	0.97	8.9	-3.3	23.7	1.4	11.5	-1.7	28.7	1.89	15.9	3.9	29.7	2	23.2	12.6	34.5	2.15
Wujin	2008	6.2	-6.9	22.3	0.68	2.1	-6.9	13.7	3.83	2.8	-7.5	19.1	0.7	11.3	1.4	23.4	1.13	15.9	7.3	28.7	2.53	22.3	11.7	33.5	4.2
Wujin	2009	4.8	-3.6	14.3	2.39	2.6	-5.8	13.8	1.27	7.8	1.2	25.8	4.14	10.1	0	26.3	1.41	16.5	2.1	28.6	1.48	22	10.5	32.4	1.63
Wujin	2010	6.7	-2.2	21.3	1.05	3.9	-5.8	18.7	0.62	6.3	-4.8	21.5	4.03	8.8	-2.8	26.2	4.27	12.8	3.4	24.7	4.52	21.3	12.9	30.9	2.57
Wujin	2011	4.7	-4.5	14.5	0.84	0.1	-5.3	7.3	0.34	5.1	-5	21.1	0.81	9.3	-0.5	22.3	1.41	16.3	2.4	31.8	0.83	22.1	11	36.7	1.59
Wujin	2012	4.2	-4.8	15.2	3.14	3.4	-5.6	10.4	1.13	3.2	-4.6	9.5	2.25	9.1	-2.1	24.8	3.23	17.8	7.2	30.8	2.68	21.9	13.9	32.7	2.66
Jintan	2005	7.2	-5.4	20.0	54.3	1.9	-6.8	10.0	37.8	3.5	-4.7	14.8	82.8	9.1	-2.4	24.2	54.9	18.2	5.9	34.3	83.8	21.2	12.1	33.2	62.5
Jintan	2006	3.9	-6.3	15.2	21.3	4.4	-4.7	18.3	120.6	4.7	-4.2	19.6	74.5	11.3	-0.1	24.7	22.9	16.7	4.9	31.7	189.3	21	12.7	32.7	82.6
Jintan	2007	5.4	-5.2	14.6	9.7	3.7	-4.6	13.2	38.9	8.9	-2.8	23.2	49.5	11.5	-1.6	29.6	67.3	15.9	3.9	30.6	74.7	23.2	13.5	33.7	74.3
Jintan	2008	6.8	-2.6	14.5	45.0	1.7	-7.6	13.9	130.3	2.6	-8.0	19.9	27.6	11.4	1.4	22.9	27.9	15.8	6.5	28.4	65.4	22.3	11.1	33.3	92.2
Jintan	2009	5.9	-7.2	23.3	15.9	2.5	-8.2	13.4	40.5	7.6	1.9	25.1	130.5	9.9	0.1	24.9	66.3	16.6	2.0	29.6	54.9	22.2	10.5	35.0	48.6
Jintan	2010	4.7	-4.5	14.6	71.9	3.7	-5.7	18.2	26.7	6.2	-4.9	21	100.2	8.7	-3.4	24.8	130.8	12.7	3.3	24.8	140.8	21	11.4	32.5	56.2
Jintan	2011	6.3	-5.0	21.7	34.7	-0.2	-9.5	7.7	17.3	5.0	-3.7	20.7	24.6	9.0	-1.9	21.1	42.2	16.2	2.9	31.9	30.0	21.7	10.2	36.5	56.8
Jintan	2012	4.3	-5.4	14.0	21.1	3.1	-6.7	11.1	37.5	3.1	-4.5	9.7	69.0	9.1	-1.5	25.8	110.2	17.9	7.6	29.4	63.0	21.7	13	32.6	90.6
Jintan	2013	4.0	-5.5	13.6	97.3	3.1	-6.0	17.4	15.7	5.8	-4.3	17.9	100.2	10.8	-1.0	30.5	53.2	15.5	1.7	31.8	29.6				
Xinghua	2003	4.2	0.8	8.4	25.2	1.4	-1.6	5.8	45.3	4.9	1.7	9.1	44.5	8.5	5.1	13.1	97.1	14.4	10.8	18.7	153.8	19.2	15.4	23.6	174.9
Xinghua	2004	6.4	3.6	10.1	18.8	2.2	-0.8	6.3	37.9	7	2.9	12.8	7.4	9.1	4.9	14.3	26.2	15.6	10.9	21.4	52.6	20.2	16.1	25.2	55.6
Xinghua	2005	3	1.2	7.2	25.6	1.2	-1.7	5.3	24.9	2	0.3	5	67.2	8	3.6	13.2	58.4	16.8	11.8	22.8	63.2	20.4	16	25.4	41.1
Xinghua	2006	5	1.7	9.4	8.6	3.5	1.3	6.5	87.7	3.6	1.3	7.1	47.7	9.5	5.4	15	15.6	15.3	11.2	20.4	91.8	19.9	16.1	24.7	78.5

Yandu	2002	4.8	-6.9	16.5	58.4	4.2	-4.7	18.2	11.1	6.1	-2.4	17.6	32.4	10.9	1.8	24.6	54.7	15.1	7.2	27.2	52.1	18.4	11.2	33.2	151.2
Yandu	2003	3.5	-3.7	15.0	15.1	2.0	-8.2	14.5	35.5	4.5	-3.9	17.6	56.4	8.0	-0.4	26.6	101.9	14.0	5.6	26.9	88.8	17.9	8.5	30.1	32.7
Yandu	2004	5.6	-7.7	18.5	18.2	1.0	-8.2	12.2	23.5	5.7	-5.5	18.4	5.4	7.9	-3.5	25.0	24.0	14.3	2.0	32.4	42.8	18.9	7.6	31.2	59.3
Yandu	2005	1.8	-5.8	15.1	20.9	0.4	-9.1	8.3	26.9	1.4	-6.7	12.0	54.9	6.5	-4.1	22.8	41.7	15.4	3.7	31.4	42.0	19.4	8.0	31.6	39.3
Yandu	2006	4.0	-5.0	13.9	17.5	2.7	-6.2	12.3	33.9	2.7	-6.8	16.3	32.5	8.6	-2.7	22.0	5.3	14.1	3.0	30.2	47.4	18.9	9.2	33.0	108.1
Yandu	2007	5.5	-3.2	13.1	82.1	2.2	-5.1	11.3	9.2	6.3	-5.1	19.4	30.5	8.9	-3.1	25.4	59.0	14.2	2.1	27.5	21.0	21.2	8.3	33.2	62.9
Yandu	2008	4.0	-10.2	19.5	24.8	1.1	-5.3	12.2	61.1	1.9	-6.1	17.8	4.4	8.9	-0.2	22.7	30.6	14.0	4.2	26.4	60.9	19.6	9.3	32.8	69.3
Yandu	2009	3.1	-5.5	11.4	36.2	1.1	-9.1	14.0	9.0	6.0	-0.6	25.0	36.7	8.1	-3.4	26.3	40.6	14.4	0.8	26.7	30.0	19.9	8.6	33.5	124.8
Yandu	2010	4.2	-6.2	20.2	24.5	1.6	-8.8	19.9	0.6	4.3	-5.9	18.8	86.4	6.6	-4.2	24.2	54.5	10.8	0.8	21.9	95.0	19.4	9.6	31.5	84.3
Yandu	2011	3.2	-5.0	12.5	18.8	-1.6	-9.3	6.9	0.1	3.0	-6.2	19.2	24.7	7.3	-2.1	21.1	20.7	14.0	1.0	30.4	10.3	20.0	10.2	33.3	60.3
Yandu	2012					1.5	-6.9	10.0	0.5	1.8	-7.5	9.6	10.1	6.9	-4.2	22.1	82.4	15.5	4.2	29.2	57.6	20.8	12.2	31.5	22.1
Dongtai	2001	4.4	1.8	7.7	109.6	3.3	0.7	6.5	14.0	4.5	1.6	8.5	13.0	9.0	4.9	13.7	5.0	13.5	9.7	18.8	20.8	21.1	16.7	26.9	16.3
Dongtai	2002	4.9	2.2	8.1	59.6	4.3	0.5	9.6	6.0	6.0	2.3	11.5	5.0	10.9	7.3	15.4	15.0	15.4	11.7	19.4	49.7	18.3	15.0	22.7	147.2
Dongtai	2003	4.2	0.7	8.6	23.7	1.5	-1.8	5.9	9.0	4.8	1.7	9.0	13.0	8.2	4.7	12.8	12.0	13.9	10.1	17.8	126.5	18.3	14.9	23.0	26.5
Dongtai	2004	6.3	3.3	10.1	21.6	2.1	-0.8	6.2	6	6.8	2.6	12.5	7.0	8.4	4.1	13.9	10.0	15.2	10.2	20.9	50.9	19.7	15.3	24.8	73.5
Dongtai	2005	2.4	-1.2	7.4	22.2	0.6	-2.7	5.3	7.0	1.9	-0.5	5.0	15.0	7.4	2.6	12.7	12.0	15.9	10.4	21.7	65.5	19.8	14.7	25.3	34.7
Dongtai	2006	4.9	1.1	9.9	9.1	3.3	0.6	6.8	16.0	3.2	0.1	7.1	14.0	9.1	4.3	15.3	7.0	14.5	10.1	18.7	80.4	19.3	14.9	23.3	87.9
Dongtai	2007	5.8	2.4	9.6	31.7	2.9	-0.4	7.2	4.0	7.1	3.2	12.5	8.0	9.6	5.9	14.5	11.0	14.3	9.2	20.1	46.1	21.4	16.3	27.5	145.9
Dongtai	2008	4.5	0.2	10.0	22.0	1.3	-1.2	4.6	14.0	2.1	-2.1	7.5	5.0	9.4	5.0	15.4	8.0	14.4	10.0	18.8	37.4	20.2	15.2	26.6	60.7
Dongtai	2009	3.6	0.8	6.9	48.7	1.6	-1.8	6.5	7.0	6.8	3.9	10.2	15.0	8.6	4.4	13.5	10.0	15.0	9.9	20.2	32.0	20.4	15.0	26.6	4.0
Dongtai	2010	4.9	1.0	10.6	32.3	2.2	-1.6	7.2	8.0	4.8	1.9	8.8	11.0	7.1	3.8	11.7	15.0	11.3	7.4	14.9	139.7	19.8	15.5	24.5	52.7
Dongtai	2011	3.5	0.4	8.0	23.5	-1.1	-4.7	3.5	4.0	3.5	0.0	9.0	8.0	7.7	3.0	13.4	7.0	14.4	9.0	19.9	18.8	20.4	15.3	25.9	57.0
Dongtai	2012	3.1	0.0	6.6	72.0	2.0	-1.4	6.1	7.0	2.3	-0.6	6.0	11.0	7.5	3.8	12.0	13.0	16.0	11.4	20.7	67.2	20.8	16.4	26.3	16.5
Hongze	2001	3.4	0.9	6.6	73.4	2.3	0.2	5.2	82	4.2	1.4	7.5	41.5	9.3	5.5	14.1	7.1	14.7	10.3	19.6	29.6	22.3	18	27.3	6.6
Hongze	2002	3.8	1.6	6.9	61	3.9	0.7	8.8	23.3	6.5	2.5	11.9	28	11.3	7.6	15.7	45.8	15.7	12.2	20.1	63.3	18.8	15.7	22.9	98.7
Hongze	2003	3.9	0.9	7.6	18.7	0.7	-2.3	4.8	21.9	4.2	1.5	7.7	54.7	8.4	4.9	12.7	87.6	14.4	11.2	18.4	88.7	19.5	15.9	23.9	51.1
Hongze	2004	5.5	3	9.1	22.7	1.8	-1.1	5.6	26	6.7	2.8	11.5	7.3	9.2	5	14.2	23.9	16.1	11.4	21.7	25.3	20	15.9	25	49.6
Hongze	2005	2.2	-1.45	6.9	21.2	0.2	-3.3	4.6	19.3	1.3	-1.5	4.7	45.4	7.4	2.6	13.2	48.2	16.8	11.1	23.4	63.2	20	15.2	25.3	53.1
Hongze	2006	3.5	-0.5	8.5	27.5	2.2	-0.4	5.5	51	3	-0.2	7	36.7	9.7	4.7	15.8	15.2	15.4	10.7	20.8	54.3	19.6	15.1	25	110.5

Hongze	2007	5.5	2.4	9.2	19.8	2.2	-1.4	6.8	7	7	3.1	12.2	27.8	9.9	6	14.6	66.4	15.2	10.2	21	26.8	22	17	27.9	94.8
Hongze	2008	4.1	0	9.4	17.1	0.5	-1.9	3.8	57.9	2	-2.1	7.4	5.1	10.3	5.1	16.1	23.4	14.8	10.5	20.1	52.2	21.3	16.4	27	113.6
Hongze	2009	3	-0.2	6.5	30.9	1.2	-2.1	5.9	7.3	6	2.9	9.8	32	8.8	4.5	14	29.6	15.7	10	21.4	26.7	20.7	15.2	26.5	77.1
Hongze	2010	4.7	0.5	10	17.7	1.9	-2.3	7.2	0.5	4.6	1.1	8.8	111.9	7.3	3.9	11.7	52.6	11.8	7.4	16.6	113.4	20	15.5	25	50.5
Hongze	2011	3	-0.3	7.1	35.4	-1.4	-5.4	3.3	0.7	3.4	-0.7	8.7	44.8	0.6	3.7	13.9	23.3	15.6	9.9	21.6	16.6	21	15.8	26.4	40.5
Hongze	2012	2.2	-0.4	5.1	76.2	1.6	-1.6	6	2.9	2.3	-0.9	6.3	17.4	7.9	4.1	12.5	90.6	16.8	12.3	22.3	31.8	22	17.1	27.1	16.6
Jiangyan	2004	4.6	-3.6	16.8	0.78	2.6	-6.3	13.3	1.6	7	-4.5	18.9	0.5	8.4	-6.6	24.8	1.4	15.1	2.3	32.4	2.6	19.9	9.5	32.8	3
Jiangyan	2005	6.6	-5.6	20.1	0.6	1.6	-6.9	9.5	1	2.4	-5.2	14.5	2.5	7.5	-2.9	23	1.4	16.3	4.1	31.5	2.8	19.9	9.4	32.5	1.6
Jiangyan	2006	3.1	-5	15.7	5.3	3.5	-5.2	14.3	3.8	3.6	-4.8	17.3	1.8	9.5	-0.7	22	0.6	15	5.1	30.6	2.9	19.7	10.2	32.3	2.6
Jiangyan	2007	5.3	-4.3	14	0.3	3.4	-3	11.8	0.3	7.5	-3.1	21.6	1.2	10.1	-2.5	28.3	2.3	14.9	4.7	28.9	1.2	22.1	10.6	33.3	1.6
Jiangyan	2008	6.5	-2	14.1	1.2	1.6	-5	12.8	2.4	2.7	-4.3	18.6	0.4	10	1.3	22.8	1	15.1	4.5	27.4	1.4	20.9	10.9	32.9	5.2
Jiangyan	2009	5.5	-8	21.8	0.8	2.3	-7.8	14	1	7.1	0.2	25.8	3	9.3	0.1	26.2	1.4	15.6	2.1	28.6	1.5	21	7.9	34.5	2.4
Jiangyan	2010	4.4	-3.7	13.1	2	2.9	-6.4	19.9	0.5	5.4	-4.7	20.8	4	7.8	-4	25.5	3.1	11.9	2.9	23.4	6	20.3	10.8	32.3	2
Jiangyan	2011	5.9	-4.6	20.9	1.3	-0.3	-7.4	6.8	0.2	4.4	-5.8	19.9	0.7	8.4	-0.5	20.9	1	15.2	3.2	32.4	0.4	21.2	10.8	34.9	0.7
Jiangyan	2012	4.2	-3.3	13.6	-0.9	2.6	-3.5	4.5	0.3	2.6	-4.2	8.4	2.2	8.1	4.4	22.9	3.2	16.6	5.7	29.6	1.8	21.4	13.8	31.7	1.3
Jingjiang	2003	5.1	-2.9	17.2	25.6	2.7	-6.8	15.2	43.3	6.1	-2.8	20	64.7	9.5	0.3	27.9	109.1	15.3	6.4	27.3	96.3	20.2	10.7	31.6	23.2
Jingjiang	2004	7.5	-5.4	21.8	43	3.4	-6.3	13.8	67.1	8.2	-2.5	19.2	35.1	9.6	0.6	25.8	41.7	16.5	2.9	34.1	64.1	21	9.9	34.1	121.8
Jingjiang	2005	4.1	-4.6	15.9	22	2.1	-7.5	9.6	40.1	3.2	-4.9	14.6	81.2	8.8	-2.8	24.1	34.2	17.9	7.1	34.2	101.2	21.4	12.7	33.1	63.3
Jingjiang	2006	6.4	-3.1	15.5	8.7	4.5	-4.9	16.5	115.1	4.6	-4.6	18.9	64.8	11.1	-0.5	22.6	27.9	16.6	5	31.9	113.1	21.2	12.5	32.8	95.2
Jingjiang	2007	7.4	-1.9	15.4	42.3	4.3	-2.5	12.7	23.6	8.9	-2.3	22.9	43	11.5	-2	28.7	68.6	16.2	4.4	30.2	45.5	23.3	12.6	34	39
Jingjiang	2008	6.6	-7	22.2	22.5	2.4	-6.6	14.6	99.5	3.1	-4.9	18.5	19.2	11.1	2.9	23	30.8	15.8	6.7	28.6	66.3	21.9	12.3	32.5	112.2
Jingjiang	2009	5.2	-2.9	14.3	65.3	3	-7.5	14.2	38.5	7.8	1.6	25.8	107.5	10	0.8	26	61.9	16.2	2.4	28.8	52	21.7	11.6	35.1	62.6
Jingjiang	2010	7.1	-4.1	21.8	30.6	4.1	-5.5	18	12.9	`6.4	-4.2	21.9	89.2	8.5	-3.3	25.8	114.7	12.6	3.5	24.5	143.9	21	12.8	32.8	71.6
Jingjiang	2011	5.3	-3.5	13.8	25.5	0.5	-7.2	7.1	6.9	5.4	-3.8	19.6	21.6	9.3	-0.3	21.2	34.1	16.2	4.1	31.6	19.7	21.7	11.1	36.1	49.5
Jingjiang	2012	4.8	-4.5	14.8	93.4	3.6	-4.8	10.3	24.3	3.4	-4.4	9.6	60.9	9.1	-1.2	24.7	84.3	17.7	6.5	29.9	93	16.1	14.2	33	45.7
Jingjiang	2013					3.9	-4.5	16.7	10.5	5.9	-4.1	18.8	106	10.9	-0.3	30.2	30.9	15.8	4	30	24.6	21.6	12.9	32.6	128.9
